# Supplementary material for: Scalable in vitro production of defined mouse erythroblasts
Source: PLoS One. 2022 Jan 7;17(1):e0261950. doi: 10.1371/journal.pone.0261950 (PMC8741028; doi:10.1371/journal.pone.0261950)
Supplement: S1 Fig — (PDF) [file pone.0261950.s001.pdf]

S1 Figure

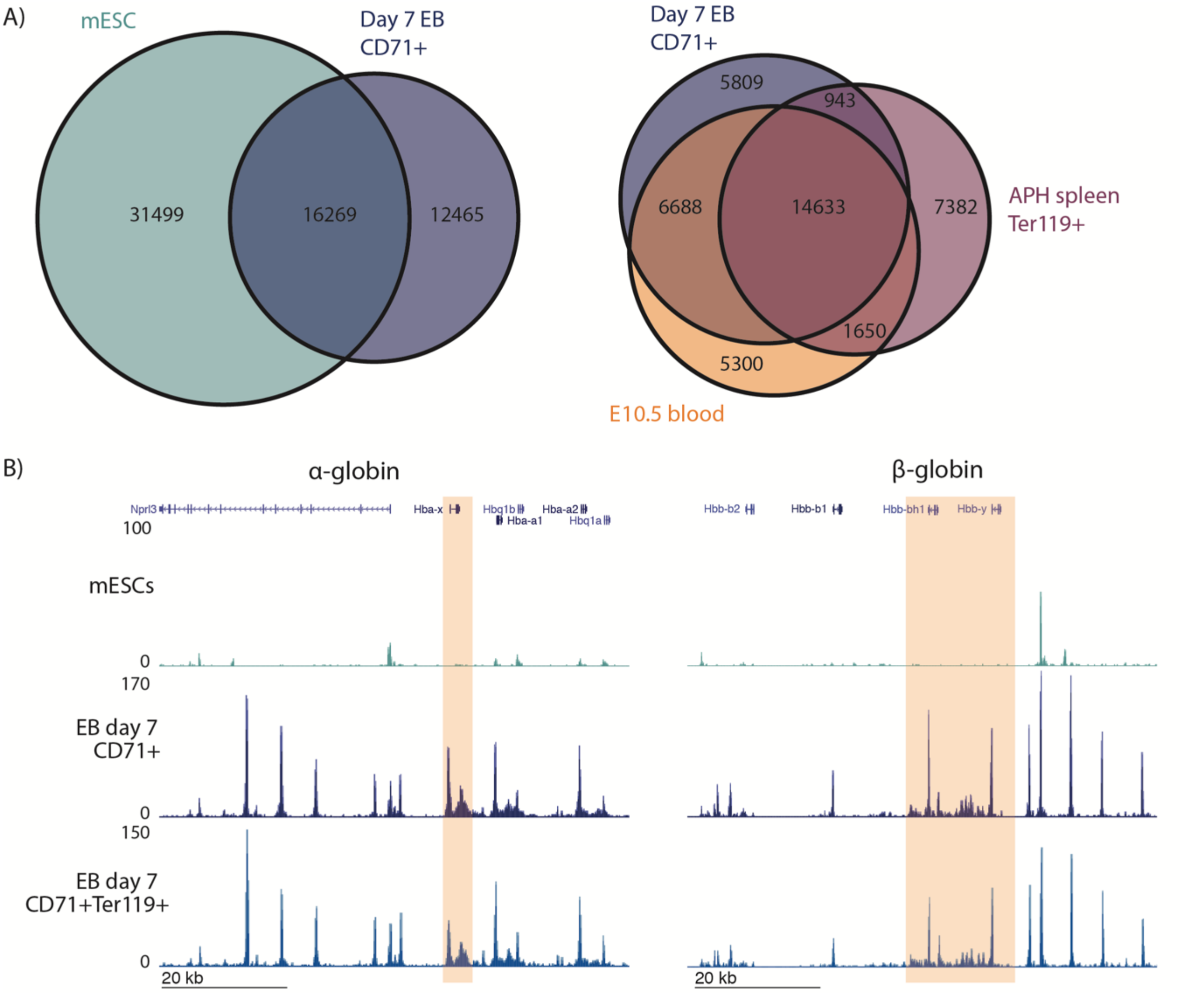

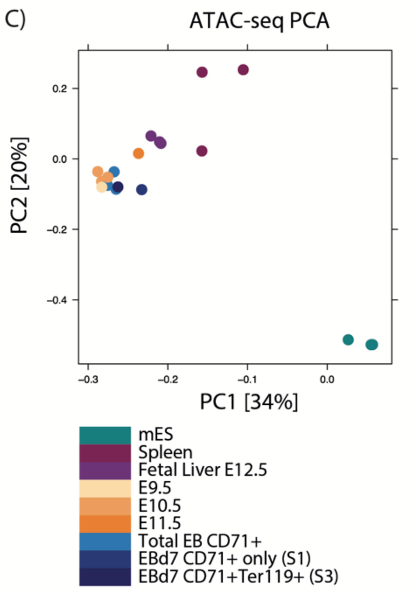

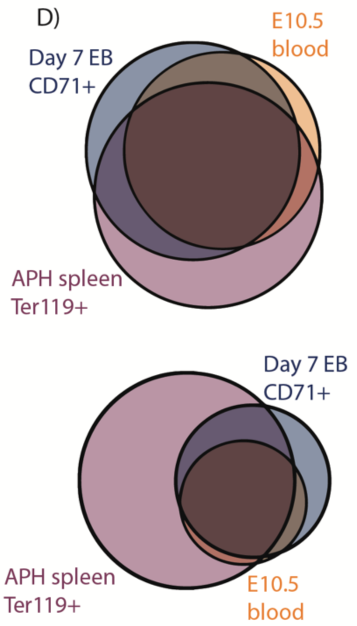

CTCF	Day 7 EB CD71+	E10.5 blood	APH spleen Ter119+
Day 7 EB CD71+	38,856	29,100	27,562
E10.5 blood	29,100	32,435	25,475
APH spleen Ter119+	27,562	25,475	38,383

EB-only	E10.5 -only	spleen-only	Intersetion
6,658	2,324	9,810	24,464

H3K4me3	Day 7 EB CD71+	E10.5 blood	APH spleen Ter119+
Day 7 EB CD71+	11,147	8,591	9,430
E10.5 blood	8,591	8,670	8,394
APH spleen Ter119+	9,430	8,394	18,366

EB-only	E10.5 -only	spleen-only	Intersetion
1,717	14	8,660	8,329

S1 Fig: More detailed ATAC-Seq analyses of CD71 sub-populations (CD71+ S1, CD71+/Ter119+ S3) and CTCF and H3K4me3 ChIP-Seq data confirm the primitive nature of these populations.

A) Venn diagrams of overlapping accessible regions between tissue datasets. Peaks were included in the analysis (if present) in data from three biological replicate tracks. Left: mESCs vs EB-derived cells; Right: EB-derived, primitive and definitive erythroid cells.

B) RPKM-normalised ATAC-seq track averaged for three replicates of double positive CD71+Ter119+ cells compared to data from total CD71+ populations, both derived from day 7 EBs.

C) PCA plot of genome-wide ATAC-seq peaks, as in Figure 2B, with addition of primitive E9.5, E10.5 and E10.5 blood, the EB-derived CD71+ (S1) and CD71+/Ter119+ (S3) populations.

D) Venn diagrams of overlapping ChIP-seq peaks between tissue datasets. Peaks included in the analysis are from three biological replicate tracks for APH Ter119+ spleen and E10.5 blood and duplicates from EB day 7 CD71+ population. Upper diagram: CTCF ChIP-seq peaks with tables containing the detailed dataset from all three cell types. Lower diagram: H3K4me3 ChIP-seq peaks presented similarly as above.
