# Supplementary material for: Scalable in vitro production of defined mouse erythroblasts
Source: PLoS One. 2022 Jan 7;17(1):e0261950. doi: 10.1371/journal.pone.0261950 (PMC8741028; doi:10.1371/journal.pone.0261950)
Supplement: S2 Fig — (PDF) [file pone.0261950.s002.pdf]

S2 Figure

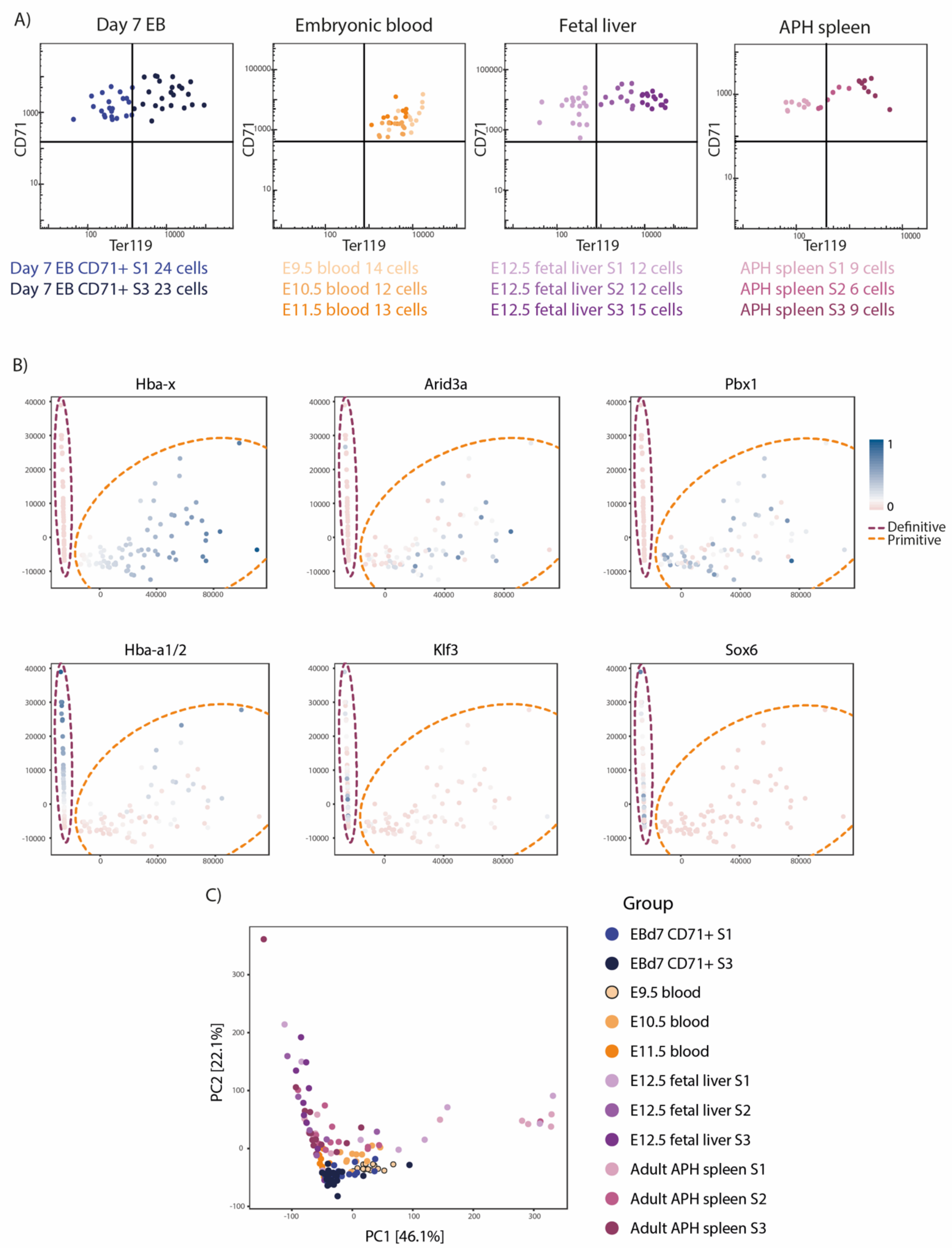

A)
Day 7 EB
Embryonic blood
Fetal liver
APH spleen
CD71
Ter119
Day 7 EB CD71+ S1 24 cells
Day 7 EB CD71+ S3 23 cells
E9.5 blood 14 cells
E10.5 blood 12 cells
E11.5 blood 13 cells
E12.5 fetal liver S1 12 cells
E12.5 fetal liver S2 12 cells
E12.5 fetal liver S3 15 cells
APH spleen S1 9 cells
APH spleen S2 6 cells
APH spleen S3 9 cells
B)
Hba-x
Arid3a
Pbx1
Hba-a1/2
Klf3
Sox6
Definitive
Primitive
C)
Group
EBd7 CD71+ S1
EBd7 CD71+ S3
E9.5 blood
E10.5 blood
E11.5 blood
E12.5 fetal liver S1
E12.5 fetal liver S2
E12.5 fetal liver S3
Adult APH spleen S1
Adult APH spleen S2
Adult APH spleen S3
PC1 [46.1%]
PC2 [22.1%]

S2 Fig: Further characterisation of the day7 EB-derived CD71 erythroid cells based on their single cell (Biomark) expression assay confirm their primitive nature.

A) CD71/Ter119 Immunophenotyping plots for FACS-sorted single cells used in RT-PCR experiment (Biomark). The corresponding labels and cell numbers for each population are shown below each plot.

B) Expression for six representative genes demonstrating enrichment in either primitive (top) or definitive (bottom) cells. Relative expression level is represented by the accompanying colour scale plotted on the PCA arrangement from Figure 3B.

C) PCA of housekeeper-normalised expression levels for non-globin probes. Clustering is observed between EB-derived (EBd7) and embryonic erythroid cells (E9.5, E10.5, E11.5 blood) away from definitive red cell populations as indicated by the 'Group' legend.
