# Supplementary material for: Scalable in vitro production of defined mouse erythroblasts
Source: PLoS One. 2022 Jan 7;17(1):e0261950. doi: 10.1371/journal.pone.0261950 (PMC8741028; doi:10.1371/journal.pone.0261950)
Supplement: S3 Fig — (PDF) [file pone.0261950.s003.pdf]

S3 Figure

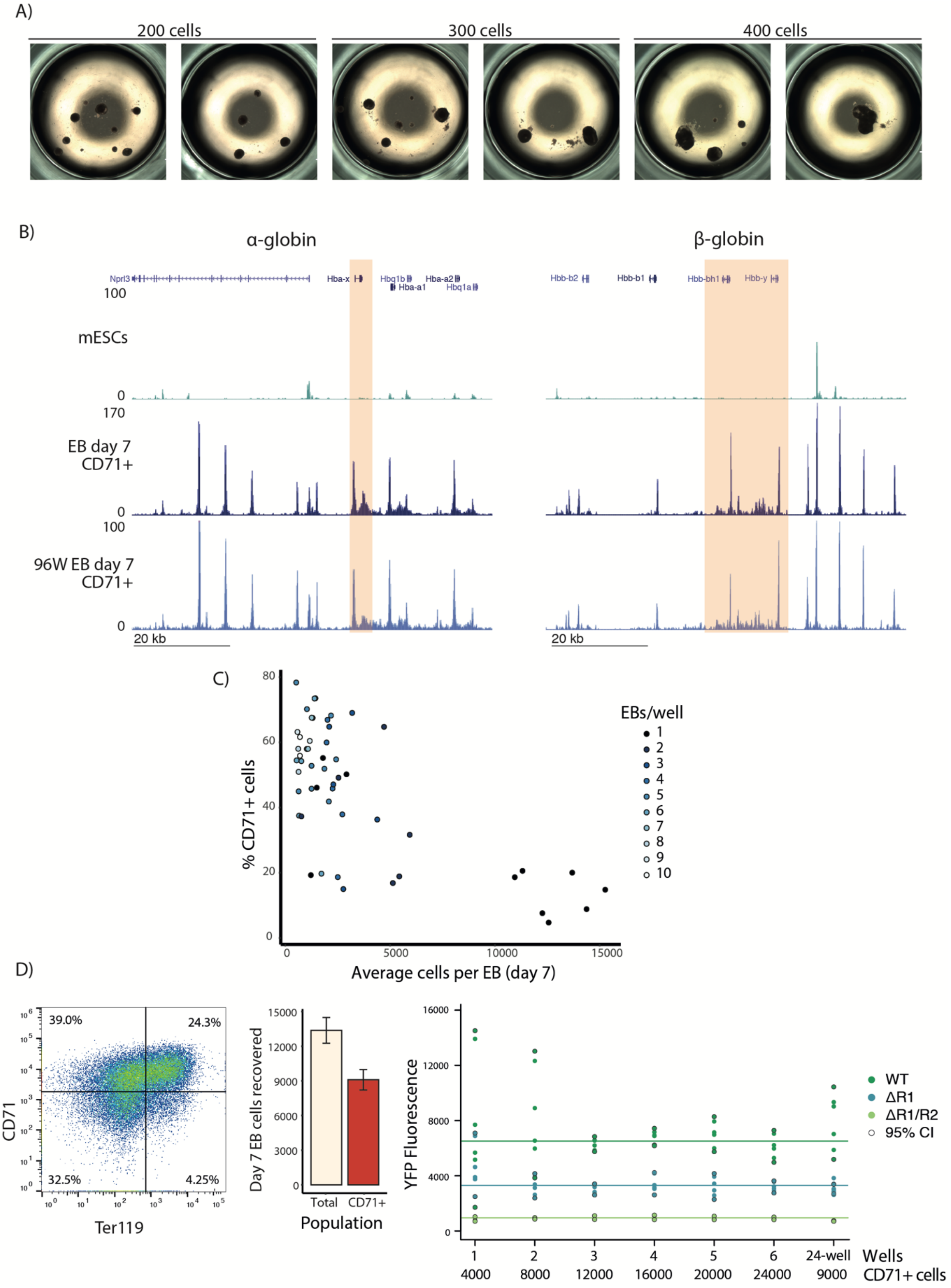
A)
200 cells
300 cells
400 cells
B)
α-globin
β-globin
Nprl3
Hba-x
Hbq1b
Hba-a1
Hba-a2
Hbq1a
Hbb-b2
Hbb-b1
Hbb-bh1
Hbb-y
mESCs
EB day 7 CD71+
96W EB day 7 CD71+
20 kb
C)
% CD71+ cells
Average cells per EB (day 7)
EBs/well
D)
CD71
Ter119
39.0%
24.3%
32.5%
4.25%
Day 7 EB cells recovered
Total
CD71+
Population
YFP Fluorescence
WT
ΔR1
ΔR1/R2
95% CI
Wells
CD71+ cells
24-well

S3 Fig: Detailed documentation of the 96- and 24-well plate miniaturisation of the EB culture shows the optimal cell number required based on the optimal EB growth and CD71+ output as determined by the larger standard 10cm dish cultures.

A) Images of six miniaturised day 7 EB wells, each plated in parallel with the optimal range of 200-400 mESCs in 200 μl differentiation media. Smaller numbers and larger-sized EBs form as cell numbers increase.

B) RPKM-normalised ATAC-seq track averaged for three replicates of cells derived from day 7 96-well EBs compared to data from mESCs and the day 7 EB-derived CD71+ population from 10 cm-dish cultures.

C) Plot of the percentage of CD71+ cells against the average number of cells per EB for individual wells plated with a range of 100-800 mESCs per well. Points are coloured by the number of individual EBs per well. More efficient production of CD71+ cells is observed when high numbers of EBs/well of smaller EB size is achieved (top left of the graph).

D) Immunophenotype, EB population cell count and readout variability plots for EBs differentiated in a 24-well plate format, each plated with 1000 mESCs in 1 ml differentiation media. Cell counts and YFP measurements were made from at least four single wells each by flow cytometry. The YFP readout for the 24-well plate (last data point, as indicated) is added to the 96-well plate readout data (as in Figure 5E).
