# Supplementary material for: Scalable in vitro production of defined mouse erythroblasts
Source: PLoS One. 2022 Jan 7;17(1):e0261950. doi: 10.1371/journal.pone.0261950 (PMC8741028; doi:10.1371/journal.pone.0261950)
Supplement: S1 Table — (PDF) [file pone.0261950.s004.pdf]

S1 Table

gRNA used for CRISPR-Cas9 genome editing

**Modification**	**gRNA Sequence**
YFP-tagged α-globin	CCTCTGGACAAATTCCTTGC
	CGTTAAGCTGCCTTCTGCGG
ΔR1	GTGACCATAAGTTGATTAG
	GTACATACGCCGGGCGTGG
ΔR2	TACCTCCAAGGTTTTGCTC
	GCCGTGACACTTCATGCTCA
D3839 (double nickase)	GGCCACTGGGGGCGCCATTC
	CGCCATTAAAAGGTCCTGCT
	TTGAGCAAACTGAGGTCCTG
	AGGCCTCTGCTACCCTCTGG
