# Supplementary material for: Scalable in vitro production of defined mouse erythroblasts
Source: PLoS One. 2022 Jan 7;17(1):e0261950. doi: 10.1371/journal.pone.0261950 (PMC8741028; doi:10.1371/journal.pone.0261950)
Supplement: S2 Table — (PDF) [file pone.0261950.s005.pdf]

S2 Table

Primer pairs used for expression analysis by RT-PCR

Gene	Primer	Sequence
Hba-a1/2	Forward	CTGGGGAAGACAAAAGCAAC
	Reverse	GCCGTGGCTTACATCAAAGT
Hba-x	Forward	ATGCGGTTAAGAGCATCGAC
	Reverse	GGGACAGGAGCTTGAAGTTG
Hbb-b1/2	Forward	ACGATCATATTGCCCAGGAG
	Reverse	ATGGCCTGAATCACTTGGAC
Hbb-bh1	Forward	TGGACAACCTCAAGGAGACC
	Reverse	ACCTCTGGGGTGAATTCCTT
Hbb-y	Forward	TGGCCTGTGGAGTAAGGTCAA
	Reverse	GAAGCAGAGGACAAGTTCCCA
Rn18s	Forward	GTAACCCGTTGAACCCCATT
	Reverse	CCATCCAATCGGTAGTAGCG
pb4.2	Forward	GCTCCAACCCACACATTTCT
	Reverse	GCATCTCTTTCCTCCACTGC
CD71	Forward	TCCGCTCGTGGAGACTACTT
	Reverse	ACATAGGGCGACAGGAAGTG
Nprl3	Forward	ATGTTCGCCAGTGTTGTTGA
	Reverse	GCTCTTCAGGTACCCCTTCC
Mpg	Forward	CTTCTCCAGCCCAGAGGAC
	Reverse	ATGCCTCAGTCTCCACAATG
Rhbdf1	Forward	CGGCCACTTGGTGATATCTT
	Reverse	ATCCTAGTGCCCCAGACCTT
Snrnp21	Forward	GAGGTAATGCCTGTGGTCGT
	Reverse	GGTCAGATGGTATGTCCGCC
