# Supplementary material for: Scalable in vitro production of defined mouse erythroblasts
Source: PLoS One. 2022 Jan 7;17(1):e0261950. doi: 10.1371/journal.pone.0261950 (PMC8741028; doi:10.1371/journal.pone.0261950)
Supplement: S3 Table — (PDF) [file pone.0261950.s006.pdf]

# S3 Table

## Biomark TaqMan assays

| Gene Name | Assay ID |
|---|---|
| *Abcg4* | Mm00507247_m1 |
| *Aqp1* | Mm01326466_m1 |
| *Aqp8* | Mm00431846_m1 |
| *Arid3a* | Mm00492248_m1 |
| *Ccr2* | Mm00438270_m1 |
| *Cited2* | Mm00516121_m1 |
| *Cox6c* | Mm00835813_g1 |
| *Cpeb4* | Mm01193440_m1 |
| *Ddx21* | Mm00497941_m1 |
| *Foxh1* | Mm00514851_m1 |
| *H2afy* | Mm01337623_gH |
| *Hba-a1/a2* | Custom (IDT) |
| *Hba-x* | Mm00439255_m1 |
| *Hbb-bh1* | Mm00433932_g1 |
| *Hbb-bh2* | Mm01273444_g1 |
| *Hbb-bt/bs/b1/b2* | Custom (IDT) |
| *Hbb-y* | Mm00433936_g1 |
| *Hbq1a* | Mm00731011_s1 |
| *Hbq1b* | Mm02747875_s1 |
| *Hspe1* | Mm00434083_m1 |
| *Il9r* | Mm00434313_m1 |
| *Klf3* | Mm00492956_m1 |
| *Lmo4* | Mm00495373_m1 |
| *Mbd2* | Mm00521967_m1 |
| *Mpg* | Mm00447872_m1 |
| *Mpo* | Mm01298424_m1 |
| *Myc* | Mm00487804_m1 |
| *Myl6* | Mm02342525_g1 |
| *Nprl3* | Mm01193449_m1 |
| *Oaz1* | Mm01611061_g1 |
| *Pbx1* | Mm04207617_m1 |
| *Podxl* | Mm00449829_m1 |
| *Pogz* | Mm00554475_m1 |
| *Prtn3* | Mm00478323_m1 |
| *Rhbdf1* | Mm00711711_m1 |
| *Rhd* | Mm00456910_m1 |

| | |
|---|---|
| *Sec61g* | Mm01613099_g1 |
| *Selk* | Mm00785961_s1 |
| *Smarca4* | Mm01151944_m1 |
| *Snrnp25* | Mm00547218_m1 |
| *Sox4* | Mm00486320_s1 |
| *Sox6* | Mm01274768_m1 |
| *Tmem14c* | Mm00481276_m1 |
| *Vamp5* | Mm00444144_m1 |
| *Zbtb7a* | Mm00657132_m1 |
