# Supplementary material for: Scalable in vitro production of defined mouse erythroblasts
Source: PLoS One. 2022 Jan 7;17(1):e0261950. doi: 10.1371/journal.pone.0261950 (PMC8741028; doi:10.1371/journal.pone.0261950)
Supplement: S4 Table — (PDF) [file pone.0261950.s007.pdf]

S4 Table

Custom BioMark assays

Assay	Left primer	Right primer	Internal oligo
Hba-a1/a2	TGAAGCCCTGGAAAGGATGT	TGAAATCGGCAGGGTGGT	TGGATCCCGTCAACTTCAAGCTCCT
Hbb-bt/bs/b1/b2	CCGATGAAGTTGGTGGTGAG	ACATGCAGCTTGTCACAGTG	CCTGGGCAGGCTGCTGGTTG
