# Supplementary material for: Scalable in vitro production of defined mouse erythroblasts
Source: PLoS One. 2022 Jan 7;17(1):e0261950. doi: 10.1371/journal.pone.0261950 (PMC8741028; doi:10.1371/journal.pone.0261950)
Supplement: S1 Methods — (DOCX) [file pone.0261950.s008.docx]

**S1 Methods**

**Genome engineering: CRISPR-Cas9 strategies and mESC transfections**

We used CRISPR-Cas9 targeting strategies for the generation of various mESC models used in this study. For guide RNA (gRNA) sequences, see Table 1. To generate the YFP-tagged α-globin mESCs, two gRNAs were cloned using a BbsI-mediated enzymatic ligation assisted by nucleases method (ELAN)^1^ into a single PX462 vector (pSpCas9n(BB)-2A-Puro), a gift from Feng Zhang. The α-globin gene (Hba-a1) was replaced by the same gene modified by the insertion of a 2A self-cleaving peptide sequence followed by mVenus coding sequence after the final exon following a homology directed repair (HDR) approach. This HDR α-2A-YFP-NLS sequence was synthesised commercially (Thermo Fisher), then cloned into the pBluescriptSK+ vector (Addgene). For generation of enhancer deletion R1 (DelR1) and R2 (DelR2) mESCs, same strategy was followed; Two sgRNAs that flank the enhancer sequence were designed and cloned into a pSpCas9(BB)-2A-GFP (pX458) vector, a gift from Feng Zhang (Addgene plasmid #48138; http://n2t.net/addgene:48138; RRID:Addgene_48138) or a modified vector with the GFP tag exchanged for an mRuby cassette (pX458-Ruby). For generation of HS3839 deletion (D3839) mESCs, sgRNAs were designed to flank a sequence encompassing the two CTCF sites (at HS-38: TACCCTCTGGTGGC and at HS-39.5: CTGGCCACTGGGGG separated by around 1.5kb). Four guides for a CRISPR/Cas9 nickase strategy were cloned into p335 (Cas9-D10A) modified vector containing a neomycin selectable marker. An HDR vector of 2991 bp was designed such that a fragment encompassing both CTCF sites an the intervening 1447bp sequence was flanked by homology arms of 750bp. sgRNA recognition sequences outside the homology arms were also included and resulted in the release of the HDR sequence from the donor vector upon co-transfection with sgRNA/Cas9 plasmids. Protospacer Adjacent Motif (PAM) sequences were mutated where necessary to avoid cutting the HDR plasmid. CTCF consensus sequences were replaced by ScaI sites (AGTACT) for ease of screening. Modified sequences were checked using Sasquatch tools for undesirable creation of potentially active hypersensitive sites in erythroid cells.^2^

For YFP-tagged α-globin mESCs generation, cells were co-transfected with gRNAs and HDR vectors at 1:1 molar ratio using TransIT-LT1 reagent (Mirus) according to manufacturer’s instructions. Selection with puromycin (Sigma-Aldrich, 1 μg/ml) was started 48 hours after transfection and removed 24 hours later. Surviving colonies were genotyped by PCR. For the other models. mESC Transfections were performed using the Neon electroporation system (Invitrogen), according to the manufacturer’s instructions. A transfection protocol optimized for mESCs (3 pulses of 1400V/10ms) whereby 10^6^ mESCs were resuspended in buffer R and electroporated with a total of 5 μg plasmid DNA (2.5ug/gRNA for DelR1 mESCs,) and 11ug (1.25ug/gRNA and 6ug for HDR vector for D3839). After electroporation, DelR1 targeted cells were cultured for 48-72 hours prior to FACS sorting the eGFP-Ruby expressing population, which was then seeded at clonal density on gelatinized 10cm dishes for at least 6 days. D3839 cells were directly plated on a gelatinized 10cm dish at clonal density and targeted clones were selected for neomycin resistance (G418 at 200μg/ml) 24h to 72h post transfection and left to grow for at least 6 days. Colonies were picked into 96-well plates and screened for mutations by PCR and Sanger sequencing.

**EB differentiation**

24-48 h prior to differentiation, cells were induced by passaging into base media (Iscove’s modified Dulbecco’s medium (IMDM), 1.4x10^-4^ M monothioglycerol (Sigma-Aldrich) and 50 U/ml penicillin-streptomycin (Thermo Fisher)) supplemented with 15% heat-inactivated fetal calf serum (ΔFCS, Thermo Fisher) and 1000 U/ml LIF.

For EB generation, cells were disaggregated by trypsinisation and quenched in base media (as above) supplemented with 10% ΔFCS. Differentiation media was prepared fresh on the day of differentiation by supplementing base media (as above) with 15% ΔFCS, 5% protein-free hybridoma medium (PFHM-II, Thermo Fisher), 2 mM L-glutamine (Thermo Fisher), 50 μg/ml L-ascorbic acid (Sigma Aldrich), 3x10^-4^ M monothioglycerol and 300 μg/ml human transferrin (Sigma Aldrich). Unless otherwise stated, cells were plated in either triple vent petri dishes (Thermo Fisher) or flat-bottom 96-well plates (Thermo Fisher) at the following densities: for 10 cm dishes, 3x10^4^ cells were added to 10 ml differentiation media; for 96-well plates, 250 cells were added to 200 μl differentiation media. EBs were left to differentiate for up to seven days without disruption except for gentle manual shaking every few days to disrupt potential EB aggregation and/or attachment to the dishes.

EBs from 10 cm dishes were harvested by collection of the entire plate contents into falcon tubes, spinning at 1000 rpm for 5 minutes before and after a single PBS wash step to ensure complete recovery. After PBS removal, EBs were disaggregated in 0.25% trypsin (0.3 ml per 10 cm dish) for 3 minutes at 37°C with continuous manual shaking to prevent sedimentation at the bottom of the tube. Trypsin was quenched with an equal volume of FCS and a single-cell suspension obtained through tituration. Cells were collected by spinning at 1200 rpm for 5 minutes and resuspended as needed. EBs from 96-well plates were processed similarly with adjustment of volumes for washes and trypsinisation and use of multichannel pipette and pooling of replicas when necessary.

EBs from 96-well plates were prepared for analysis by a performing a single PBS wash and disaggregation in a V-bottom 96-well plate. Whole EBs were transferred and washed using a multichannel pipette, resting tips on the kink of the V-bottom plate to avoid accidental EB suction during media removal steps. Disaggregation was performed using 50 μl 0.25% trypsin per well and quenched with an equal volume of FCS. Single-cell suspensions were obtained by pipetting up and down 20 times with the multichannel pipette. Wells were topped up to 300 μl and put directly for analysis by flow cytometry or first stained with fluor-conjugated antibodies in 20 μl staining buffer (PBS with 10% FCS; 500 μl per 10^7^ cells), as required.

**Erythroid population isolation from EBs**

CD71+(high) cells were isolated by magnetic column separation (LS Column, Miltenyi), according to the manufacturer’s instructions. Briefly, cells were labelled with anti-mouse CD71-FITC (eBioscience 11-0711-85; 1:200) in staining buffer (PBS with 10% FCS; 500 μl per 10^7^ cells) for 20 minutes at 4°C, rolling, then washed by adding staining buffer (1 ml per 10^7^ cells) and spinning. After supernatant removal, cells were incubated with MACS anti-FITC separation microbeads (Miltenyli; 10 μl per 10^7^ cells) in ice-cold separation buffer (PBS plus 0.5% bovine serum albumin (BSA) and 2 mM EDTA; 90 μl per 10^7^ cells) for 15 minutes at 4°C, rolling, and washed by adding separation buffer (1 ml per 10^7^ cells) and spinning.

Bead-labelled cells were resuspended in 500 μl cold separation buffer and added to a pre-equilibrated LS column. The negative fraction was washed through with two flushes of 3 ml cold separation buffer and the positive fraction collected by forcing cells from the column in 5 ml separation buffer. After spinning and supernatant removal, cells were resuspended in staining buffer as needed for downstream processing. Population purity and selection efficiency were determined by flow cytometry.

**Flow cytometry Antibodies**

FITC Rat Anti-Mouse CD71 eBioscience 11-0711-85 (1:200)

OR APC Anti-Mouse CD71 eBioscience 17-0711-80 (1:2000)

PE Rat Anti-Mouse Ter119 BD Pharmingen 553673 (1:200)

PE-Cy7 Anti-Mouse c-kit eBioscience 25-1171-82 (1:200)

Hoechst 33258, Pentahydrate (bis-Benzimide) Invitrogen H3569 (1:10000)

**RNA expression analysis by RT-PC**

RT-PCR was performed with Fast SYBR Green Master Mix (Thermo Fisher) and data normalised to the 18S ribosomal gene using the ΔC_T_ method. RNA quality was routinely checked by Tapestation analysis (Agilent) and reverse transcriptase enzyme negative controls were included to confirm adequate DNase treatment was performed. Primer pairs used for expression analysis by RT-PCR are in Supplemental Table 2.

**Single-cell expression analysis: Biomark**

Fetal livers were dissected from embryos at embryonic day 12.5 and disaggregated in ice cold staining buffer (0.2 % BSA and 5 mM glucose in 1x PBS). Cells from spleens of phenylhydrazine APH-treated adult mice^3^ were collected by manual tissue disruption in PBS supplemented with 10% FCS. To obtain embryonic erythroid cells, pregnant female mice were dissected at 9.5, 10.5, and 11.5 days after observation of a vaginal plug. Embryos including yolk sac material were individually collected into tubes containing PBS and manually disrupted to promote erythroid cell release from the circulation. Spleen and embryo-derived cells were collected by centrifugation and resuspended in staining buffer, then treated in the same manner as the fetal liver tissue.

ChromePure Rabbit IgG (Jackson, 015-000-003) was added to a final concentration of 200μg/ml to block Fc receptors. Cells were stained with Ter119:APC (BD; 1:200), CD71:PE-Cy7 (Biolegend; 1:1500), CD117:BB700 (BD; 1:100) and Lin:FITC (equal parts CD41, B220, CD3e, Mac-1 and Gr-1, all BD FITC conjugated) on ice for 45 min in the dark. Cells were washed 2x with 1ml ice cold staining buffer then resuspended in FACS running buffer (staining buffer plus 5 mM EDTA). Immediately before sorting, Hoechst (Invitrogen) was added at 1:10000 dilution).

185 single erythroid cells were isolated by index-FACS: 16 cells each from mouse embryos at E9.5, E10.5 and E11.5; 42 cells each from spleens of APH-treated adult mice and 48 cells each from E12.5 embryos and embryoid bodies, and deposited into wells of a 96-well plate containing 5.1 μl lysis buffer (5 μl 2x reaction buffer from SuperScript™ III One-Step RT-PCR with Platinum™ Taq kit (Thermo Fisher), 0.1 μl SUPERase In™ RNase Inhibitor (Thermo Fisher)). Plates were sealed, briefly centrifuged and frozen at -20 °C. TaqMan assay mastermix was made by pooling 6.6 μl of 43 TaqMan assays (see SupplementalTable 3) and adding 376 μl TE buffer. Plates were defrosted and 4.9 μl amplification mix was added to each well (1.2 μl TE buffer (Invitrogen), 2.5 μl TaqMan assay mastermix, 1.2 μl RT/Taq enzyme mix from SuperScript™ III One-Step RT-PCR with Platinum™ Taq kit (Thermo Fisher)) except for one well containing a cell from embryoid bodies, in which Platinum Taq was substituted for RT/Taq mix to serve as a no-RTase control. cDNA synthesis and sequence-specific preamplification was then carried out (reverse transcription at 50 °C for 15 min; RTase inactivation at 95 °C for 2 min; specific target amplification by 22 cycles of 95 °C for 15 s then 60 °C for 4 min). An RNA standard curve was also prepared at this point using the same mastermix and amplification program (see below). Plates were stored at -20 °C until analysis. Pre-amplified cDNA was diluted 5x with TE buffer and analysed using Universal PCR Master Mix (Applied Biosystems) and individual Taqman gene expression assays (Life Technologies, Supplemental Table 3), on the Biomark System (Fluidigm) using two 192.24 Dynamic Arrays as per manufacturers protocol, except that elongation time was increased from 60 s to 75 s to accommodate longer custom gene-expression assays for Hba-a1/2 and Hbb-bt/s/1/2 (Supplemental Table 4).

To prepare RNA standards, T7 polymerase was used to *in vitro* transcribe RNA from cDNA clones of Cox6c, Sec61g, Oaz1, Hba-a1, Hba-x, Hbb-bt, Hbb-y and Hbb-bh1. RNA was run on a 6% acrylamide TBE-urea gel (Life Technologies) and bands corresponding to full-length transcript were isolated from the gel. RNA was extracted by incubating crushed gel pieces with elution buffer (500 mM Ammonium acetate, 1 mM EDTA-KOH pH 8.0) for 3 hours at room temperature before ethanol precipitation, resuspension in TE buffer and quantification using RNA Quantifluor assay (Promega) and RNA Tapestation (Agilent). Each RNA was diluted to 16.8 pM concentration and the 8 transcripts were pooled, aliquoted and stored at -80 °C. When performing reverse transcription and pre-amplification on sorted single cells, one aliquot of RNA standard was also defrosted, and diluted 10x. Five more serial dilutions were created by diluting 6x in TE buffer, then 2 μl was taken from each of the six standard curve dilutions and pre-amplified using the same mastermix as used for single cells (see above).

Data analysis was performed using Python (v3.5.2; Matplotlib v2.0.2; Numpy v1.14.5; Pandas v0.23.3; scikit-learn v0.19.2; Scipy v1.1.0). C_T_ values beyond the limit of detection or marked as “Fail” by the instrument were set to the limit of detection value (C_T_ = 40). 21 cells were excluded from further analysis, 15 due to > 80% of assays being undetected and 6 due to outlier C_T_ values. Raw C_T_ values were transformed into expression space by calculating 2^-C_T_. Three housekeeping genes (Cox6c, Myl6 and Sec61g) were run on both 192.24 chips. The expression of these three genes was scaled to a range of 0 to 1, then the mean of the three scaled expression values was calculated for each cell and used to normalise expression values for the remaining 21 assays on each chip. This normalisation was performed separately for the two chips before merging the data together for analysis.

**References**

1. Cost GJ. Enzymatic ligation assisted by nucleases: simultaneous ligation and digestion promote the ordered assembly of DNA. *Nature Protocols*. 2007;2(9):2198–2202.

2. Schwessinger R, Suciu MC, McGowan SJ, et al. Sasquatch: predicting the impact of regulatory SNPs on transcription factor binding from cell- and tissue-specific DNase footprints. *Genome Res.* 2017;27(10):1730–1742.

3. Spivak JL, Toretti D, Dickerman HW. Effect of phenylhydrazine-induced hemolytic anemia on nuclear RNA polymerase activity of the mouse spleen. *Blood*. 1973;42(2):257–266.
